# The pathogenesis, diagnosis, and treatment of chronic endometritis: a comprehensive review

**DOI:** 10.3389/fendo.2025.1603570

**Published:** 2025-06-12

**Authors:** Xinyang Yan, Jiao Jiao, Xiuxia Wang

**Affiliations:** ^1^ Center of Reproductive Medicine, Shengjing Hospital of China Medical University, Shenyang, China; ^2^ Shenyang Reproductive Health Clinical Medicine Research Center, Shengjing Hospital of China Medical University, Shenyang, China

**Keywords:** antibiotic therapy, chronic endometritis, female infertility, endometrial receptivity, immune dysregulation, microbial dysbiosis

## Abstract

Chronic endometritis (CE) is a subtle but persistent inflammatory disorder of the endometrium that is often underdiagnosed due to its asymptomatic or non-specific presentation. The etiology of CE primarily involves microbial infections and immune dysregulation, often accompanied by microbial dysbiosis. Diagnosis relies on histopathological examination, especially the identification of stromal plasma cells, alongside hysteroscopic findings and microbiological testing, though standardization remains lacking. Mechanistically, CE-induced infertility stems from altered immune cell profiles, impaired endometrial receptivity, aberrant decidualization, dysbiosis of the endometrial microbiota, and abnormal uterine peristalsis. Aberrant gene expression and hormone receptor dysregulation further disrupt the implantation window. This review summarizes current understanding of the diagnostic criteria, pathogenic mechanisms, and therapeutic strategies for CE, emphasizing its crucial role in infertility and the need for standardized clinical management.

## 1 Introduction

Endometritis is categorized into acute and chronic forms. Acute endometritis typically presents with sudden onset of symptoms such as fever, lower abdominal pain, and abnormal vaginal discharge, primarily associated with acute infections (1). Pathologically, it is characterized by tissue edema, hemorrhage, and infiltration of polymorphonuclear leukocytes in the endometrial stroma. Studies suggest that transient acute endometritis is not significantly associated with infertility (1). In contrast, chronic endometritis (CE) is a persistent inflammatory condition of the endometrium, often asymptomatic or presenting with mild, non-specific symptoms such as increased vaginal discharge and pelvic discomfort (2, 3). Due to its lack of distinctive clinical features, CE is frequently overlooked in gynecological practice.

However, with the advancement of assisted reproductive technologies (ART), increasing evidence indicates a high prevalence of CE among infertile women, particularly those experiencing repeated implantation failure (RIF), recurrent spontaneous abortion (RSA), and unexplained infertility (UI), with reported incidences of 57.5% (4), 56% (5) and 56.8% (6), respectively. CE has thus garnered significant attention in the field of reproductive medicine (7, 8). Although the exact etiology of CE remains unclear, it is often associated with bacterial infections, with the hallmark pathological feature being the infiltration of plasma cells in the endometrial stroma (9). The specific mechanisms by which CE contributes to female infertility are not fully understood, but current research primarily focuses on its impact on endometrial receptivity and embryo implantation (10, 11). This review aims to provide a comprehensive overview of CE and its implications for female infertility.

## 2 Diagnosis of chronic endometritis

CE is a chronic inflammatory condition of the endometrium, often asymptomatic or presenting with non-specific clinical manifestations such as abnormal uterine bleeding, pelvic pain, dyspareunia, and vaginal discharge, with abnormal uterine bleeding being the most common symptom (12–14). Peripheral blood leukocyte counts and serum C-reactive protein levels, typical inflammatory markers, are not specific for CE (15). Therefore, the diagnosis of CE relies on endometrial histopathological examination, hysteroscopy, and microbiological testing.

### 2.1 Histopathological examination: the gold standard for CE diagnosis

Histopathological examination remains the gold standard for diagnosing CE. The primary pathological features include endometrial surface mucosal edema, separation of epithelial cells and stroma, increased stromal cell density, and plasma cell infiltration in the stroma (16). The presence of plasma cells in the stroma is the most specific and sensitive diagnostic criterion. Typical CE plasma cells exhibit large cell bodies, a high nuclear-to-cytoplasmic ratio, basophilic cytoplasm, and a “spoke-wheel” heterochromatin pattern (17). However, both traditional hematoxylin and eosin (H&E) staining and immunohistochemical staining for CD138 (syndecan-1), a sensitive marker for plasma cells, have limitations in diagnosing CE, including dependence on endometrial samples, variability in staining, observer subjectivity, inconsistent timing of sample collection during the menstrual cycle, and unclear clinical significance of minimal plasma cell infiltration (18, 19).

### 2.2 Hysteroscopy evaluation

Hysteroscopy provides direct visualization of the uterine cavity, allowing for a detailed examination of the endometrial surface. CE exhibits characteristic hysteroscopic findings, including diffuse hyperemia (with a “strawberry” appearance due to white central spots), focal hyperemia, punctate hemorrhages, stromal edema, and the presence of micro-polyps (diameter <1 mm). However, there is no consensus on the diagnostic criteria for CE via hysteroscopy, leading to variability in reported incidence rates. Liu et al. (20) developed a scoring system for hysteroscopic diagnosis of CE, assigning points for various features such as diffuse hyperemia (4 points), punctate hemorrhages (2 points), focal hyperemia (2 points), dilated endometrial vessels (2 points), micro-polyps (1 point), polyps (1 point), and a history of repeated intrauterine insemination failure (2 points), with a total score of 14. The optimal cutoff value for diagnosing CE was >2 points based on ROC curve analysis and the Youden index. While hysteroscopy provides valuable information, its diagnostic accuracy is influenced by the operator’s subjective judgment and the quality of the equipment. Therefore, hysteroscopy should not replace histopathological examination, and a combination of both methods enhances diagnostic accuracy (21, 22).

### 2.3 Microbiological testing

Given the inflammatory nature of CE and the effectiveness of antibiotic treatment, pathogen detection is crucial for diagnosis and targeted therapy. The uterine cavity is not a sterile environment, and a balanced microbiota is essential for endometrial development and embryo implantation. Dysbiosis, characterized by an overgrowth of pathogenic bacteria or a reduction in beneficial bacteria, can lead to endometrial inflammation (23). CE cannot be diagnosed through lower genital tract microbial cultures alone. Instead, a double-sheath sampling catheter is recommended to avoid contamination from the vagina and cervix, allowing for the collection of endometrial tissue or lavage fluid for microbial culture. Studies have shown that selecting antibiotics based on culture results significantly improves CE cure rates (24). However, microbial cultures have limitations, including the inability to culture certain pathogens, such as Chlamydia trachomatis, Mycoplasma, and Neisseria gonorrhoeae, the fact that only 1% of bacteria can be cultured, susceptibility to environmental contamination, and long turnaround times for results. The diagnostic challenges of CE stem from its heterogeneous presentation and the limitations of individual modalities. A comparative analysis of current diagnostic modalities, including their strengths and limitations, is provided in **Table 1**.

**Table 1:** Diagnostic modalities for chronic endometritis.

Diagnostic Method	Key Features	Advantage	Limitation
Histopathological Examination	Gold standard; plasma cell infiltration in endometrial stroma is diagnostic.	High specificity and sensitivity for plasma cell detection.	Dependent on sample quality, observer subjectivity, and timing in menstrual cycle.
Hysteroscopy	Direct visualization of uterine cavity; features include hyperemia, micro-polyps.	Provides real-time imaging; complements histopathology.	Subjective interpretation; lacks standardized diagnostic criteria.
Microbiological Testing	Identifies pathogens (e.g., *E. coli*, *Mycoplasma*) via endometrial cultures.	Guides targeted antibiotic therapy; improves treatment outcomes.	Limited by inability to culture certain pathogens; risk of contamination.

## 3 Etiology and risk factors of chronic endometritis

The hallmark of CE is the presence of numerous plasma cells in the functional and basal layers of the endometrium. The exact etiology remains unclear, but abnormal immune cell distribution often indicates an underlying immune response. Potential causes include exogenous infections, autoimmune diseases, and tissue damage. Given the effectiveness of antibiotic therapy in most cases, CE is widely believed to be associated with microbial infections. Common pathogens include Escherichia coli, Enterococcus faecalis, Streptococcus agalactiae, and Mycoplasma (25–28). Cicinelli et al. (29) conducted endometrial microbial cultures in 438 women diagnosed with CE via hysteroscopy and 100 non-CE controls. The results showed a 73.1% positive culture rate in CE patients, with common bacteria accounting for 58%, Ureaplasma for 10%, and Chlamydia for 2.7%, compared to only 5% in non-CE women. Additionally, CE may be associated with intrauterine adhesions, multiple endometrial polyps, intrauterine device (IUD) placement, and endometriosis. The incidence of CE is significantly higher in women with these conditions compared to the general population (30–34). For instance, the incidence of CE in women with intrauterine adhesions is 35.40% (32), while those with multiple endometrial polyps have a twofold increased risk of CE (33). The incidence of CE following IUD placement is 30.00% (35, 36), and women with endometriosis have a 2.7-fold higher risk of CE (29). Pain or abnormal uterine bleeding in these patients may be partially attributed to CE.

## 4 Mechanisms of chronic endometritis-induced infertility

### 4.1 Immune dysregulation in the endometrium

The human endometrium contains various immune cells, including natural killer (NK) cells, macrophages, and T cells (37–41). The composition and density of these immune cell populations fluctuate cyclically during the menstrual cycle and pregnancy. Peripheral blood NK cells predominantly express the CD56dimCD16+ phenotype, whereas endometrial NK (uNK) cells are primarily CD56brightCD16-. CD56dim cells are more cytotoxic, while CD56bright cells are the main source of immunoregulatory cytokines (42). CD16+ cells exhibit stronger cytolytic activity than CD16- cells. In normal women, uNK cells increase significantly during the secretory phase of the menstrual cycle and early pregnancy. Studies have found that women with RSA have a lower percentage of CD56brightCD16- uNK cells and a higher percentage of CD56dimCD16+ cells during early pregnancy, which may contribute to increased cytotoxicity and impaired trophoblast invasion, leading to a higher susceptibility to early pregnancy loss (43, 44).

In non-pathological endometrium, B cells are primarily located in the basal layer, accounting for less than 1% of endometrial leukocytes. In CE patients, abnormal immune cell distribution is observed, characterized by B lymphocyte infiltration into the stroma and glandular epithelium, increased CD3+ T cells, elevated CD8+ T cells and Foxp3+ regulatory T cells (Tregs), and reduced CD56+CD16- NK cells. This altered immune milieu is detrimental to embryo implantation and is a significant factor in repeated implantation failure (45). Furthermore, microbial antigens such as lipopolysaccharide (LPS) induce the expression of selectin E on uterine microvascular endothelial cells, secretion of CXCL13, and production of CXCL1 by endometrial epithelial cells, leading to selective extravasation of B cells into the endometrial stroma and subsequent differentiation into plasma cells (46). These plasma cells express various immunoglobulin subclasses (IgM, IgA1, IgA2, IgG1, and IgG2), with IgG2 being the most abundant. The excessive production of mucosal antibodies may negatively impact endometrial receptivity (ER), thereby impairing embryo implantation (47).

### 4.2 Impairment of endometrial receptivity

Embryo quality and endometrial receptivity (ER) are critical factors for successful pregnancy. ER refers to the ability of the endometrium to allow embryo implantation during a specific period, known as the “implantation window,” which typically occurs between days 20 and 24 of the menstrual cycle. The synchronization of ER with embryonic development is crucial for successful implantation. This process involves the dynamic and orderly expression of numerous genes. In CE patients, the gene expression profile related to endometrial receptivity is altered. Di Pietro et al. (48) compared the gene expression profiles of 16 CE patients and 10 non-CE women during the implantation window, focusing on genes involved in inflammation, proliferation, and apoptosis. The results showed upregulation of insulin-like growth factor binding protein-1 (IGFBP-1), BCL-2, and BAX, and downregulation of IL-11, CCL-4, IGF-1, and CASP8 in CE patients. These molecular changes significantly impact the embryo implantation process.

IGFBP-1, produced by endometrial stromal cells, regulates reproductive processes through the IGF/IGFBP system (49, 50). Increased IGFBP-1 levels reduce IGF-1 and IGF-2, affecting endometrial decidualization. BCL-2, an anti-apoptotic gene, is highly expressed during the follicular phase, decreases during the luteal phase, and is minimally expressed during menstruation (51). In contrast, BAX and CASP8 are pro-apoptotic genes. The imbalance between anti-apoptotic and pro-apoptotic factors in CE patients may disrupt tissue remodeling during embryo implantation and placental development, leading to endometrial hyperplasia and the formation of micro-polyps (52). IL-11, produced by stromal and epithelial cells, has multifunctional anti-inflammatory effects and is crucial for trophoblast invasion, embryo implantation, and stromal cell decidualization (53). CCL-4, a chemokine, recruits NK cells and macrophages from peripheral blood to the endometrium (54). The downregulation of CCL-4 in CE patients may contribute to implantation failure.

### 4.3 Dysbiosis of the endometrial microbiota

Recent studies have shown that the uterine cavity is not sterile, and alterations in the endometrial microbiota are associated with CE. Kanako et al. (55) analyzed the endometrial microbiota of infertile women using 16S rRNA gene sequencing and found that Lactobacillus predominates in the endometrial microbiota. However, the relative abundance of Lactobacillus was significantly lower in CE patients (1.89%) compared to non-CE women (80.7%). In contrast, CE patients had higher levels of Gardnerella, Prevotella, and anaerobic cocci. Another study found that in infertile patients with a history of RIF, the CE group exhibited a loss of Lactobacillus dominance in the uterine cavity, while Corynebacterium and Mycoplasma hominis were more frequently detected compared to the non-CE group (56, 57). Women with a non-Lactobacillus-dominant microbiota have significantly lower implantation, pregnancy, ongoing pregnancy, and live birth rates compared to those with a Lactobacillus-dominant microbiota (58). The endometrial microbiota may influence the phenotype and function of immune cells, which recognize microbial presence through receptors, establishing a host-microbe interaction that promotes an implantation-friendly microenvironment and tolerance to non-sterile semen passing through the uterine cavity. However, the precise mechanisms underlying the interaction between the endometrial microbiota and immune cells remain unclear (59).

### 4.4 Abnormal decidualization

Decidualization is a process in which the endometrium undergoes extensive morphological, expressive, and secretory changes to support embryo implantation and development (60). It involves stromal cell proliferation and differentiation, increased glandular secretion, NK cell aggregation, and spiral artery remodeling (61, 62). Decidualization is regulated by the sequential actions of estrogen and progesterone and their receptors. The decidua plays a protective role against oxidative stress, promotes trophoblast invasion, and maintains pregnancy. It also facilitates gas, nutrient, and metabolite exchange between the mother and fetus. Impaired decidualization is associated with implantation failure and pregnancy complications (63). The decidua produces various hormones, growth factors, and cytokines, including prolactin (PRL), corticotropin-releasing factor (CRF), IGFBP-1, and IL-15, which are important markers of decidualization (64). Evidence suggests that decidualization is impaired in CE patients. Real-time PCR analysis of decidual markers in cultured endometrial stromal cells revealed significantly lower PRL/IGFBP-1 expression in CE patients compared to non-CE controls. Additionally, CE patients exhibited abnormal upregulation of estrogen receptor (ER) α/β and progesterone receptor (PR) A/B in stromal cells, as well as increased ERα and ERβ expression in glandular cells (65). In normal endometrium, ER expression in stromal and glandular cells is downregulated during the mid-luteal phase. The abnormal upregulation of ER in CE patients may disrupt the hormonal regulation of endometrial stromal decidualization (66).

### 4.5 Aberrant gene and receptor expression

In some CE patients, upregulation of nuclear markers such as Ki-67, associated with estrogen and progesterone receptors and cell proliferation, has been observed in endometrial epithelial and stromal fibroblasts. Additionally, anti-apoptotic genes (BCL2 and BAX) are upregulated, while local inflammatory genes (IL11 and CCL4) related to embryo receptivity are downregulated. These changes result in delayed endometrial differentiation during the mid-secretory phase, altering the implantation window and impairing embryo implantation (19). Abnormal expression of sex hormone receptors in CE patients weakens the effects of progesterone on endometrial stromal cells (ESCs), reducing their differentiation potential and enhancing their proliferative capacity. This makes it difficult for the endometrium to initiate decidualization and disrupts the development of endometrial decidualization, ultimately affecting embryo implantation and pregnancy (66, 67).

### 4.6 Altered endometrial contraction patterns

The uterus exhibits cyclic changes in contractility throughout the menstrual cycle. Endometrial waves (EWs), originating from the myometrium, are a characteristic feature of uterine motility. Estrogen promotes myometrial contractions, while progesterone reduces myometrial contractility (68). Consequently, the amplitude, direction, and frequency of EWs vary cyclically. Antegrade contractions, predominant during the early follicular phase, facilitate the expulsion of menstrual debris. Retrograde contractions, predominant during ovulation, aid in sperm migration toward the fallopian tubes. During the luteal phase, EW activity is minimal. Chronic inflammation, as seen in CE, can alter uterine contractility, leading to hypercontractility or dyskinesia, which may contribute to uterine motility disorders and dysmenorrhea in CE patients. Studies have shown that CE patients exhibit significant differences in EW patterns compared to normal women. Specifically, CE patients have a 3.3-fold reduction in retrograde contractions during ovulation and unnecessary contractions during the mid-luteal phase, which may impair sperm migration and blastocyst implantation (68). These alterations in EW activity may contribute to adverse pregnancy outcomes in CE patients. The multifactorial pathogenesis of CE-induced infertility involves crosstalk between immune dysfunction, microbiota dysbiosis, and molecular defects in endometrial receptivity (**Table 2**).

**Table 2:** Key mechanisms linking chronic endometritis to infertility.

Mechanism	Key Findings	Clinical Implications
Dysregulation of Endometrial Immune Microenvironment	Altered immune cell distribution (e.g., increased CD8^+^ T cells, reduced uNK cells).	Impairs embryo implantation; linked to repeated implantation failure (RIF).
Impaired Endometrial Receptivity	Altered gene expression (e.g., upregulation of IGFBP-1, downregulation of IL-11).	Disrupts implantation window; reduces embryo attachment and survival.
Dysbiosis of Endometrial Microbiota	Reduced *Lactobacillus* dominance; increased *Gardnerella* and *Prevotella*.	Associated with lower pregnancy and live birth rates.
Abnormal Decidualization Process	Reduced PRL/IGFBP-1 expression; abnormal estrogen/progesterone receptor levels.	Compromises endometrial support for embryo implantation and placental development.
Aberrant Gene and Receptor Expression	Upregulation of Ki-67, BCL2, and BAX; downregulation of IL-11 and CCL4.	Delays endometrial differentiation; disrupts implantation window.
Altered Endometrial Contraction Patterns	Reduced retrograde contractions during ovulation; unnecessary mid-luteal contractions.	Impairs sperm migration and blastocyst implantation.

## 5 Therapeutic approaches and reproductive outcomes of chronic endometritis

### 5.1 Antibiotic therapy and emerging adjuvants

While antibiotic therapy forms the cornerstone of CE management (69), emerging adjuvant approaches, such as intrauterine platelet-rich plasma infusion, aim to address refractory cases and restore endometrial function (70). There is no standardized treatment protocol for CE. However, numerous studies have demonstrated that antibiotic therapy effectively eliminates plasma cells in the stroma, thereby improving pregnancy outcomes in CE patients (55, 71). The choice of antibiotics, treatment duration, and route of administration vary widely in clinical practice (72, 73). Commonly used drugs include doxycycline, metronidazole, ciprofloxacin, and levofloxacin (74). Doxycycline is often the first-line treatment due to its broad-spectrum activity against common bacteria and Mycoplasma (75, 76). Systemic antibiotic therapy is the primary treatment approach. For patients who do not respond to multiple courses of systemic antibiotics, intrauterine antibiotic administration has shown efficacy in some cases (77–79).

### 5.2 Fertility outcomes after CE treatment

Most studies indicate that treating CE improves reproductive outcomes (80). In women with a history of RIF, the live birth rate in the first IVF-ET cycle and cumulative live birth rate over three IVF-ET cycles were significantly higher in the treated CE group (32.8% and 38.8%, respectively) compared to the non-CE group (22.1% and 27.9%, respectively) (56, 79). Another study found that in women with UI, the natural pregnancy rate (PR) and live birth rate (LBR) were significantly higher in the treated CE group (PR=76.3% *vs*. 20% *vs*. 9.5%; LBR=65.8% *vs*. 6.6% *vs*. 4.8%) compared to the persistent CE and non-CE groups (6). These findings suggest that antibiotic therapy effectively treats CE and improves pregnancy outcomes. However, the optimal treatment regimen and timing of follow-up remain unclear and require further investigation. In some cases, intrauterine autologous platelet-rich plasma infusion has been successfully used to treat CE patients who did not respond to antibiotics, resulting in successful pregnancies (81).

## 6 Conclusion

Chronic endometritis (CE) is a significant yet often overlooked cause of female infertility, with its pathogenesis involving complex interactions between microbial infections, immune dysregulation, and altered endometrial receptivity. The diagnosis of CE remains challenging due to its non-specific symptoms and the lack of standardized diagnostic criteria. However, advancements in histopathological, hysteroscopic, and microbiological techniques have improved detection rates. Antibiotic therapy has shown promise in improving reproductive outcomes, though further research is needed to establish standardized treatment protocols. Understanding the multifaceted mechanisms underlying CE-induced infertility is crucial for developing effective therapeutic strategies. Future studies should focus on elucidating the molecular pathways involved and optimizing diagnostic and treatment approaches to improve fertility outcomes in affected females.
